# Effect of hearing training on hearing and speech skills in children with speech sound disorders

**DOI:** 10.1590/2317-1782/e20240008en

**Published:** 2025-02-28

**Authors:** Cristiane Dellinghausen Valim, Márcia Keske-Soares, Eliara Pinto Vieira Biaggio

**Affiliations:** 1 Universidade Federal de Santa Maria – UFSM - Santa Maria (RS), Brasil.

**Keywords:** Auditory Perception, Child, Software, Acoustic Stimulation, Speech Disorders

## Abstract

**Purpose:**

to measure the effect of Brief Computerized Auditory Training (Brief-CAT) on behavioral auditory and speech skills in children with Speech Sound Disorder (SSD).

**Methods:**

14 children, aged 6 and 9, diagnosed with SSD participated. All children presented one or more altered auditory skills in the behavioral assessment of Central Auditory Processing (CAP). They underwent six sessions of Brief-CAT. CAP's behavioral auditory skills and speech were assessed and a questionnaire was administered pre and post Brief-CAT. Inferential analysis was carried out.

**Results:**

Brief-CAT had an effect on the background figure ability for verbal sounds. The CAP tests individually showed an evolution in the number of subjects who changed their profile from “altered” to “normal”, even if not all of them had normalized the assessment. Prior speech therapy and the type of SSD had no impact on CAP results after Brief CAT. The questionnaire sustained the same results after intervention. Combining speech therapy with Brief-CAT offered greater potential for improving the phonological system (reducing absent sounds, increasing acquired sounds, and lowering SSD severity). Children with phonological disorders exhibited enhanced speech outcomes with combined Brief-CAT compared to those with motor speech disorders.

**Conclusion:**

Brief-CAT proved effective in enhancing figure-background auditory abilities in children with SSD. Associating speech therapy with Brief-CAT should be the preferred therapeutic approach as it provides greater progress. The type of SSD influenced the speech performance of children undergoing Brief-CAT.

## INTRODUCTION

Speech acquisition occurs throughout early childhood and, around the age of five, the child is expected to be able to produce the speech pattern in which he or she is socially inserted. When such acquisition does not occur as expected and alterations in its production are established, such as exchanges, omissions and/or distortions of phonemes, we have Speech Sound Disorders (SSD)(1,2). Such disorders are caused by etiological processes, both genetic and environmental, associated with neurodevelopment(3). On this developmental basis, speech processes, such as representation (auditory and somatosensory), transcoding (planning and programming) and motor execution contribute to the refinement and/or impairment of speech(1). Speech impairment in these specific processes gives rise to different types of SSD.

There are different classifications of SSD(4,5), but the most common clinical typology currently presents the distinction between Speech Delay (or Phonological Disorder - PD), Speech Errors - SE (including Phonetic Disorder and Persistent Speech Errors), and Motor Speech Disorder - MSD (and its subtypes: Motor Speech Delay - MSD, Childhood Apraxia of Speech - CAS, Childhood Dysarthria - CD, and the combination of these), differentiated according to their diagnostic markers(1).

Previously published studies relate auditory skills and linguistic skills, especially in children with SSD(6-13), highlighting that difficulties in auditory perception of complex sounds, such as speech sounds, are present in this population. This reaffirms the need for auditory assessment, both in terms of sensitivity and auditory perception. Thus, as a complementary assessment to the basic audiological assessment, the behavioral assessment of Central Auditory Processing (CAP) is used to measure performance related to the functionality of central auditory skills(14,15).

In the presence of changes in CAP behavioral tests, the result is “delay in the development of central auditory skills” (children aged up to 6:11.29 days) or a diagnosis of Central Auditory Processing Disorder (CAPD) (children over 7 years old). In both cases, the complementary speech therapy approach involves stimulating altered auditory skills(15,16).

Among the intervention strategies, Computerized Auditory Training (CAT) is an interesting therapeutic option, as the use of software, in addition to being scientifically referenced(6,9,17), is interactive and motivating. The gamification strategies used contribute to maintaining attention/motivation and therapeutic engagement(17). Thus, such intervention provides challenging listening activities (in an afferent way - bottom-up process), recreating adaptive listening possibilities, as well as the need for motor response (efferent pathways - top-down process) by these children(15).

There are studies in the national literature correlating AT with speech and language disorders(18,19), as well as in cases of PD(20,21). However, the relationship between AT and different SSDs, including MSDs, still lacks reports, mainly because it encompasses several types of disorders and does not have a greater focus on linguistic skills.

Thus, through this research we sought to study a brief therapeutic approach for children with behavioral changes in CAP tests who present SSD.

Therefore, the objective of this research was to verify the effect of Brief Computerized Auditory Training (Brief-CAT) on the behavioral auditory skills and speech of children with SSD.

## METHODS

This is a longitudinal, prospective, quantitative study of a clinical-experimental nature, approved by the Institution's Research Ethics Committee under number 68074623.0.0000.5346. All guidelines of Resolution 466/12 of the National Health Council were respected.

The initial sample consisted of 26 children recruited from a Speech Therapy teaching clinic at a Higher Education Institution. Of these, 14 completed all stages of the research. It is worth noting that the participants were undergoing treatment or were waiting in line for speech therapy at the service, and the inclusion criteria for this study were: previous diagnosis of SSD(1); tonal auditory thresholds within normal standards in both ears and all frequencies(22,23); in one or more central auditory skills altered in the behavioral assessment of CAP(14,16). Exclusion criteria were established regarding: children with previously diagnosed neurodevelopmental disorders; with musical education and/or bilingual; who had been exposed to previous auditory training; who had not completed the proposed therapeutic program and/or did not perform the reassessments within the pre-determined time for data collection.

To establish the diagnosis of the different SSD, all subjects underwent the following assessments in the speech sector of the service: Phonological Assessment (INFONO software - Phonological Assessment Instrument)(24); Expressive Vocabulary Assessment (Children's Naming Test - CNT)(25); Comprehensive Vocabulary Assessment (Auditory Vocabulary Test - AVLT)(26); Dynamic Assessment of Speech Motor Skills(27); and Orofacial Myofunctional Assessment with Scores (OMAS)(28). Based on the data obtained by INFONO, in the spontaneous naming stage, the severity of SSD was analyzed with the results of the Percentage of Correct Consonants (PCC)(29,30), classified into four levels: Mild (PCC>85%); Mild-Moderate (PCC between 65%-85%); Moderate-Severe (PCC between 50%-65%); and Severe (PCC<50%). The SSD were classified according to the aforementioned typology(1), and the sample included cases of: Phonological Disorder (PD); and Motor Speech Disorders (MSD), with the subtypes Motor Speech Delay (MSD) and Childhood Apraxia of Speech (CAS). It should be noted that cases aged over nine years were classified as having Persistent SSD(31), either PD or MSD. In addition to identifying the type of SSD of the participants, the sample was divided into two groups: children with SSD in speech therapy; and children with SSD on the waiting list.

Afterwards, the sample group was invited to participate in the study and the parents and/or guardians signed the FICF and answered the anamnesis questions. Next, a basic audiological evaluation was performed (Meatoscopy, Pure Tone Audiometry from 250Hz to 8000Hz, Speech Recognition Threshold with Figures, Percentage Index of Speech Recognition with Figures, Tympanometry and Contralateral Stapedial Acoustic Reflex Research). All should present tonal hearing thresholds within normal standards in both ears and all frequencies(22,23).

Next, the CAP Behavioral Assessment was performed, with the following tests being performed: Sequential Memory Test for Non-Verbal Sounds (SMTnVS)(32); Dichotic Digit Test (DDT)(32); Random Gap Detection Test (RGDT)(33); Monotic Listening Test for Sentences (PSI - Pediatric Speech Intelligibility)(32). All should present at least one altered auditory skill of the CAP.

In addition, parents were asked to respond to the Auditory Processing Domains Questionnaire (APDQ)(34), consisting of 52 questions divided into the following domains: auditory processing (31 items); attention (10 items) and language (11 items). This questionnaire aims to quantify, through the parents' perception, the auditory behavior of children and was applied in the waiting room by an experienced evaluator. Parents answered the questions objectively on a four-point scale. In this way, the children's auditory behavior was evaluated, scoring each question: four points if the behavior was observed most of the time; three points when observed frequently; one point if observed sometimes; and zero points when observed rarely.

After these procedures for sample composition, participants were selected for the research. As a research procedure, an intervention protocol called Brief Computerized Auditory Training (Brief-CAT) was carried out. The Brief-CAT was mediated by the use of the Escuta Ativa® software(35), involving 12 activities to stimulate the auditory skills of binaural interaction, figure-background, temporal resolution, temporal standardization, discrimination, integration and binaural separation.

The sample group underwent the Brief-CAT, which was carried out in six training sessions, twice a week, lasting 30 to 45 minutes each, held in a silent room, using an Acer - Aspire 3 (A315-53-55DD) computer and Sennheiser brand supra-auricular headphones, model HD 559.

In each session, two tasks were worked on, with different levels of difficulty (easy, medium, difficult and insane, as named by the software itself). The aim was for each child to get a minimum of 70% of the tasks correctly in order to progress in terms of difficulty and thus complete all the proposed activities. The two activities worked on per session followed the order in which the software was presented and were presented to the sample group in the same sequence.

Chart 1 was created to better visualize the Brief-CAT protocol carried out using the Escuta Ativa® Software(35), describing the tasks adopted in each session, their characteristics, main auditory skills involved, and the levels of difficulty in each of them.

**Chart 1:** Computerized Auditory Training Protocol adopted: description of each task, main auditory skills stimulated and difficulty levels per task of the Escuta Ativa® software

Session number	Task	Characteristics	Main skills	Difficulty levels
1st Session	How Many Intervals	Activity where the subject must perceive the intervals of silence between auditory stimuli such as pure tones, music and phrases.	Auditory attention and temporal resolution.	Easy, medium, hard and insane (between tones, songs and phrases).
	Which Sound Was Heard	Two similar words are presented and the subject must answer whether they are the same or different. In this last level, a competitive noise is inserted, increasing the difficulty of the task.	Auditory attention and discrimination.	Easy, medium, hard and insane.
2nd Session	Hearing and Attention	Two words are heard and it must be identified whether they are in accordance with the task statement (related to phonological awareness).	Auditory analysis and synthesis, auditory discrimination, divided attention, executive function.	Easy, medium, hard and insane.
	How many sounds	Different sounds related to different instruments are offered with the intention of identifying the amount of stimuli presented.	Temporal resolution and auditory attention.	Easy, medium, hard and insane.
3nd Session	Follow the Flute	Sequences with different sound patterns are presented and the subject must reproduce them. They differ in duration (short - long) and are played to the sound of a flute. The number of sound stimuli in a sequence varies from 3 to 5 sounds.	Temporal patterning and auditory memory.	Easy (3 sounds), medium (3 sounds), hard (4 sounds) and insane (5 sounds).
	Follow the Piano	Sequences with different sound patterns are presented and the subject must reproduce them. They differ in frequency (low - high) and are played on a piano. The number of sound stimuli in a sequence varies from 3 to 5 sounds.	Temporal patterning and auditory memory.	Easy (3 sounds), medium (3 sounds), hard (4 sounds) and insane (5 sounds).
4th Session	Follow the Sequence	The subject hears a sequence of sounds (such as animals) and must score them in the presented scenario, according to the orders requested on the screen.	Auditory memory, working memory, integration of non-verbal and verbal, executive function.	Easy, medium, hard and insane.
	Right in the Crosshairs	Dichotic stimuli are presented and the subject must identify in which ear a given word was presented. The target sounds are numbers, words and dichotic expressions.	Binaural separation and auditory attention.	Easy (digits and words), medium (words, verbs and opposition), difficult (verbs, opposition and sayings) and insane (sayings).
5th Session	Left – Right	Two words are presented in a dichotic manner, and the subject must identify which words are heard in each channel, selecting them from several similar options presented on the screen.	Binaural integration and auditory attention.	Easy, medium, hard and insane.
	Binaural	The location and distance of stimuli that are presented in different positions (right or left / far or near) must be identified.	Binaural interaction and auditory attention.	Easy, medium, hard and insane.
6th Session	Catch It If You Can (Bonus Track)	The subject must follow the rapid movement of an item on the screen, and click on it.	Visual attention, visual-manual coordination.	Easy, medium, hard and insane (with stages 1, 2 and 3).
	Follow the Rhythm (Bonus Track)	Using a predetermined song, the subject must hit the highest number of musical notes heard, pressing direction arrows on the keyboard that are differentiated by colors.	Temporal processing, processing speed.	Easy, medium, hard and insane.

As reassessment procedures, the CAP behavioral tests were used again, the APDQ was reapplied, and a new speech assessment was performed through the reapplication of the INFONO, within a maximum period of 30 days after the Brief-CAT was performed, following the same methodological standards for evaluating the results of the pre-intervention stage.

The reassessment stage was carried out by qualified professionals from the research group, who did not have access to the pre-intervention assessment data and who were not responsible for applying the Brief-CAT protocol. Thus, it can be concluded that this study has a blinded data analysis, to reduce or eliminate potential confirmation bias.

The post-intervention stage aimed to measure the effect of brief-PCT (complementary therapeutic approach) in children diagnosed with SSD and the co-occurrence of altered auditory skills in the CAP assessment.

To analyze the data, an inferential study was carried out, in which the results of the CAP behavioral assessment were measured in both quantitative and qualitative terms. The data obtained from the APDQ analysis were analyzed qualitatively. Regarding speech data, these were analyzed quantitatively.

Furthermore, the variables analyzed were: being or not in speech therapy for SSD (in attendance or on the waiting list for it) and regarding the diagnosis of SSD (PD or MSD).

Descriptive and inferential statistical analyses were performed using Sas Studio software. After testing the assumptions of normality (Shapiro-Wilk), homogeneity of variances (Levene) and independence of errors (residual graph), the CAP responses were analyzed using the Tukey test and Fisher's nonparametric test and significant differences were declared when the p value was <0.05 and trends were considered when 0.05 ≥ P ≤ 0.10. Furthermore, sample sufficiency was proven by power analysis, in which probability values ​​above 0.85 were observed for the main variables extracted from the total data set.

## RESULTS

The sample initially recruited, for convenience, was made up of 26 children. Based on the eligibility criteria and the availability to participate in the intervention proposal researched, the sample was composed of 14 children with SSD, 11 boys (78%) and three girls (22%), aged between 6 and 9 years (average of 7.3 years of age).

Regarding the type of SSD, nine children (64%) were diagnosed with PD, four of whom had Persistent PD, and five children (36%) had MSD. Of the latter, the diagnoses were SMD and CAS, with no cases of CD or association between CAS and CD, and only one child had Persistent MSD.

**Figure 1:**
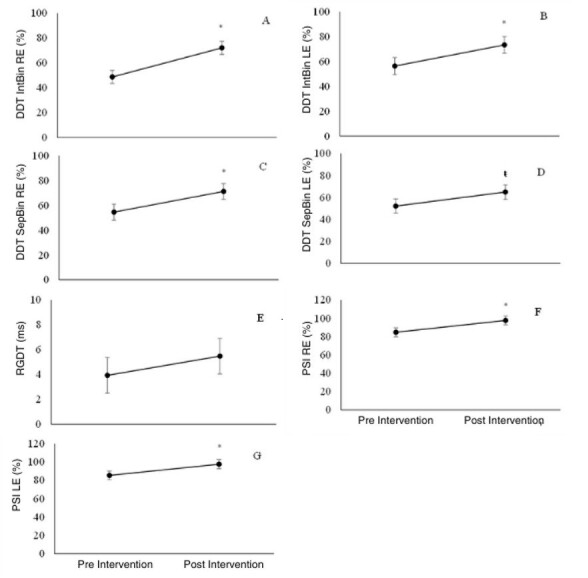
(A-G) Effect of Brief-CT on auditory skills pre- and post-intervention in children with SSD: quantitative analysis (n=14)

Figure 1 presents the effect of Brief-CAT on the results of the different CAP behavioral tests, measuring the changes in the percentages of correct answers post-intervention, regardless of the type of SSD.

**Caption:** DDT IntBin = Dichotic Digits Binaural Integration Test; DDT SepBin = Dichotic Digits Binaural Separation Test; RGDT = Random Gap Detection Test; PSI = Pediatrics Speech Intelligibility; RE = Right Ear; LE = Left Ear; % = percentage; ms = milliseconds; Pre = results from the stage before Brief-CAT; Post = results from the stage after Brief-CAT; ŧ = indicates trend between pre and post intervention; *indicates statistically significant difference between the pre- and post-intervention stages Tukey test

Table 1 presents the effect of Brief-CAT on children's responses (Normal or Altered) for each of the tests of the CAP behavioral assessment, using Fisher's Exact Test.

**Table 1:** Effect of Brief-CAT on post-intervention auditory skills in children with SSD: qualitative analysis (n=14)

Intervention	Response		p-value^1^
	Normal	Altered	
**SMTnVS**			
Pre	8 (57.1)	6 (42.9)	0.59
Post	13 (92.9)	1 (7.1)	**<0.01** *
**DDT IntBin RE**			
Pre	2 (14.3)	12 (85.7)	<0.01*
Post	7 (50.0)	7 (50.0)	0.99
**DDT IntBin LE**			
Pre	1 (7.1)	13 (92.9)	<0.01*
Post	7 (50.0)	7 (50.0)	0.99
**DDT SepBin RE**			
Pre	6 (42.9)	8 (57.1)	0.59
Post	8 (57.1)	6 (42.9)	0.59
**DDT SepBin LE**			
Pre	4 (28.6)	10 (71.4)	0.11
Post	7 (50.0)	7 (50.0)	0.99
**RGDT (ms)**			
Pre	9 (64.3)	5 (35.7)	0.28
Post	10 (71.4)	4 (28.6)	0.11
**PSI RE**			
Pre	13 (92.9)	1 (7.1)	<0.01*
Post	14 (100.0)	0 (0.0)	-
**PSI LE**			
Pre	13 (92.9)	1 (7.1)	<0.01*
Post	14 (100.0)	0 (0.0)	-

1 Probability by Fisher's Exact Test at 5% significance;

* indicates statistically significant difference between the pre and post intervention stages

**Caption:** SMTnVS = Sequential Memory Test for Non-Verbal Sounds; DDT IntBin = Dichotic Digits Binaural Integration Test; DDT SepBin = Dichotic Digits Binaural Separation Test; RGDT = Random Gap Detection Test; PSI = Pediatrics Speech Intelligibility; RE = right ear; LE = left ear; ms = milliseconds; n = sample number; Pre = results from the stage before the adopted intervention; Post = results from the stage after the adopted intervention; SEM = Standard Error of the Mean

In Table 2, the effect of Brief-CAT on the auditory skills of children with SSD was analyzed, in each test of the CAP behavioral assessment, considering whether the sample subject was attending therapy or waiting in line, using the Tukey Test.

**Table 2:** Effect of Brief-CAT on auditory skills in children with SSD with and without previous speech therapy (n=14)

Responses	Therapy		SEM^1^	p-value^2^
	Yes (n=8)	No (n=6)		
DDT IntBin RE (%)	73.69	47.15	6.22	**0.01** *
DDT IntBin LE (%)	68.43	61.27	9.13	0.59
DDT SepBin RE (%)	66.66	59.05	7.76	0.51
DDT SepBin LE (%)	59.19	57.89	8.34	0.91
RGDT (ms)	5.82	3.60	1.70	0.38
PSI RE (%)	99.31	83.06	5.82	0.08
PSI LE (%)	99.25	83.89	5.84	0.09

1 SEM = Standard Error of the Mean;

2 Probability by Tukey Test at 5% significance;

* indicates statistically significant difference between the pre and post intervention stages

**Caption:** DDT IntBin = Binaural Integration Dichotic Digit Test; DDT SepBin = Binaural Separation Dichotic Digit Test; RGDT = Random Gap Detection Test; PSI = Pediatrics Speech Intelligibility; RE = right ear; LE = left ear; % = percentage; ms = milliseconds; n = sample number; Pre = results from the stage before the adopted intervention; Post = results from the stage after the adopted intervention; SEM = Standard Error of the Mean; Yes = participating in speech therapy for SSD; No = waiting in line for speech therapy for SSD

In Table 3, the effect of Brief-CAT on the auditory skills of children was analyzed, in each test of the CAP behavioral assessment, considering the type of ssd, that is, with PD (n=9) or with MSD (n=5), also with the Tukey Test.

**Table 3:** Effect of Brief-CAT on the auditory skills of children with Phonological Disorder and Motor Speech Disorder (n=14)

Responses	SSD Type		SEM^1^	p-value^2^
	PD (n=9)	MSD (n=5)		
DDT IntBin RE (%)	72.94	47.90	6.34	**0.02** *
DDT IntBin LE (%)	72.02	57.67	9.32	0.32
DDT SepBin RE (%)	85.02	40.69	7.91	<0.01*
DDT SepBin LE (%)	73.94	43.14	8.51	0.03*
RGDT (ms)	4.44	4.98	1.74	0.83
PSI RE (%)	99.21	83.16	5.93	0.09
PSI LE (%)	99.73	83.42	5.96	0.09

1 SEM = Standard Error of the Mean;

2 Probability by Tukey Test at 5% significance;

* indicates statistically significant difference between the pre and post intervention stages

Legend: DDt IntBin = Dichotic Digit Test Binaural Integration; DDT SepBin = Dichotic Digit Test Binaural Separation; RGDT = Random Gap Detection Test; PSI = Pediatrics Speech Intelligibility; RE = right ear; LE = left ear; % = percentage; ms = milliseconds; n = sample number; Pre = results of the stage before the adopted intervention; Post = results of the stage after the adopted intervention; SEM = Standard Error of the Mean; SSD = Speech Sound Disorder; PD = Phonological Disorder; MSD = Motor Speech Disorder

Regarding the effect of Brief-CAT on the perception of guardians in relation to children's auditory behavior, such change in auditory behavior was not perceived after the intervention, according to the analysis of the APDQ responses (p-value = 0.99) performed using Fisher's Exact Test.

Regarding the effect of Brief-CAT on the perception of guardians in relation to children's auditory behavior, such change in auditory behavior was not perceived after the intervention, according to the analysis of the APDQ responses (p-value = 0.99) performed using Fisher's Exact Test.

Table 4 presents the effect of Brief-CAT on speech, that is, phonological aspects of the general phonological inventory and the severity of TSF, considering the variable history of previous speech therapy (being in speech therapy or being in line waiting for care), through the Tukey Test.

**Table 4:** Effect of Brief-CAT on speech outcomes of children with SSD with and without speech therapy

Responses	Therapy		SEM^1^	p-value^2^
	Yes (n=8)	No (n=6)		
Absent sounds (n)	1.16	6.54	1.48	**0.03** *
Partially acquired sounds (n)	1.37	1.72	0.55	0.66
Acquired sounds (n)	16.46	10.73	1.77	0.03*
Correct consonants (%)	84.08	63.04	6.93	0.06
Severity^3^	1.53	2.34	0.23	0.01*

1 SEM = Standard Error of the Mean;

2 Probability by Tukey's Test at 5% significance;

3 Severity: 1 = mild (more than 85% of consonants correct), 2 = mild/moderate (between 65 and 85% of consonants correct), 3 = moderate/severe (between 50 and 65% of consonants correct) and 4 = severe (below 50% of consonants correct);

* indicates statistically significant difference

In Table 5, the effect of Brief-CAT on speech was analyzed, regarding the phonological aspects of the general phonological inventory and the severity of SSD, according to the type of SSD (PD or MSD), as well as the other inferential analyses with the Tukey test.

**Table 5:** Effect of Brief-CAT on speech outcomes of children with different types of SSD (PD or MSD)

Responses	SSD Type		SEM^1^	p-value^2^
	PD (n=9)	MSD (n=5)		
Absent sounds (n)	1.22	6.48	1.52	**0.04** *
Partially acquired sounds (n)	1.09	2.01	0.55	0.59
Acquired sounds (n)	16.69	10.51	1.66	0.03*
Correct consonants (%)	87.04	60.08	7.07	0.03*
Severity^3^	1.36	2.50	0.25	<0.01*

1 SEM = Standard Error of the Mean;

2 Probability by Tukey's Test at 5% significance;

3 Severity: 1 = mild (more than 85% of consonants correct), 2 = mild/moderate (between 65 and 85% of consonants correct), 3 = moderate/severe (between 50 and 65% of consonants correct) and 4 = severe (below 50% of consonants correct);

* indicates statistically significant difference

**Caption:** SSD = Speech Sound Disorder; PD = Phonological Disorder; MSD = Motor Speech Disorder

## DISCUSSION

The effect of Brief-CAT was effective in improving the figure-ground ability for verbal sounds in children with SSD. When observed qualitatively and individually, all CAP tests showed progress. However, it is worth highlighting that, despite these results, the Brief-CAT, in this sample, did not have the power to adapt all the auditory skills evaluated and there was no change in the parents' perception regarding auditory behavior after such intervention. The variables “Speech therapy for SSD” and “type of SSD” did not influence the results of the behavioral assessment of CAP after Brief-CAT.

The effect of Brief-CAT on speech results was important, as it could be inferred that speech therapy when associated with Brief-CAT offered a greater possibility of adapting the phonological system, that is, a reduction in absent sounds, greater production of acquired sounds and less severity of SSD. Furthermore, children with PD obtained better speech results when performing the associated Brief-CAT, when compared to those with SSD.

When analyzing the effect of the intervention, an improvement was observed in the figure-background auditory ability for verbal sounds, measured through the DDT responses (in both test conditions and in both ears evaluated) and PSI after the adopted Brief-CAT protocol.

Different studies indicate improvements in auditory skills after auditory training, which is considered an important intervention option(17,36,37), aiming at evolution through brain neuroplasticity(38,39).

Regarding the effect of Brief-CAT on the auditory ability of figure-background for verbal sounds, it is known that this ability is associated with the analysis and synthesis of speech sounds in the presence of competing sounds. Similarly to the present study, in a research carried out with children diagnosed with CAPD, without association with SSD, the authors also used software to adapt auditory skills and observed significant improvement in this same skill(40). In research whose sample was composed of children with SSD, computerized auditory training also proved effective in improving this skill(41). It is worth remembering that figure-background ability would be related to the difficulty in separating relevant information and attending to the auditory focus of competitive noise(37). Thus, Brief-CAT may have been more efficient in this skill, considering the specificities of the chosen software, even though temporal issues are more present in the proposed stimulation.

The data from the tests that evaluated the auditory ability of temporal resolution did not show any statistically significant difference after the Brief-CT adopted in the present study. This result is different from other studies that observed improvements in this skill in children with PD after CAT using the same software(20,41) and/or other softwares that were also effective in adapting this skill in the presence of APD(40,42).

It is believed that the lack of effect of CAT on the RGDT results highlights the continued inability to auditorily perceive acoustic differences over time in the sample group of this study. It is worth highlighting that this result was possibly directly related to immaturity in the perception of speech contrasts(43). That is, such data may be related to the speech alteration of the research subjects, since temporal patterns are associated with auditory discrimination and speech segments, as such ability is crucial for the perception of small changes in intensity, duration and frequency of sounds, so important for the correct production of speech(10,18,44).

In the present study, it is possible to observe that in all tests, there was an evolution in the number of subjects who scored better (from “altered” to “normal”) after the intervention, even though the delay or CAP disorder is still characterized. The tests that presented a greater chance of modifying this profile were the SMTnVS, DDT BinInt and PSI tests, that is, the tests with a statistically significant difference. The results of the RGDT, DDT BinSep tests remained practically the same after the intervention.

Thus, the qualitative evolution of research subjects in the face of CAP behavioral assessment tests is important. However, as already mentioned, there was no improvement in all tests nor an adjustment of the auditory skills of the entire sample group, as in other national studies that used different software(6,20,21) and/or other AT modalities(37). In general, each AT protocol focuses on some auditory skills and the generalization of the effect of this intervention to other skills is still something that deserves to be analyzed more carefully.

Although the time to carry out each activity and the duration of the CAT program is not yet a consensus in the literature, it is hypothesized that one of the justifications for the results of the present study is the reduced number of sessions. In the literature, there are records of CAT protocols with eight to 40 sessions(6,9,17,20,40,45). It was decided to carry out a Brief-CAT protocol, with six sessions, thus studying a proposal with clinical applicability and lower operational cost. This choice even avoided sample loss during the execution of the Brief-CAT protocol. It is worth noting that all subjects were present in 100% of the sessions and adherence to therapy is an important variable in the rehabilitation process.

It is known that the longer the training time, the more these skills are reinforced in order to improve them(46), so new research with a greater number of sessions may be an alternative in the future, which does not invalidate the data presented here.

Furthermore, in relation to the effect of Brief-CAT on auditory skills, when analyzing the responses obtained from children undergoing speech therapy for SSD and those on the waiting list, it can be inferred that the results of the CAP behavioral battery were not influenced by this variable. This data may be a result of the therapeutic model adopted in speech therapy for TSF, in which there is no regular stimulation of the auditory skills of the CAP and the lack of stimulation in the children on the waiting list. It is known that, in the case of children diagnosed with SD, in some types of therapeutic approaches there is auditory stimulation (previously called auditory bombardment) which consists of presenting a list of words that is read by the therapist and heard by the child with the aim of stimulating auditory and visual perception of the target sound in words that are not being directly worked on in the therapy session(47). However, it is important to note that this therapeutic approach does not incorporate specific intervention in auditory skills. This strategy refers to the stimulation of auditory attention, one of the most basic skills in the hierarchy of CAP skills(15,48) and it can be suggested that such conduct has not been adopted as a standard by therapists in this public.

For the SSD type variable, the results indicate that the auditory ability of figure-background for verbal sounds is better in children with PD when compared to those with MSD. Furthermore, children with PD also present, numerically, better results in the CAP assessment, when compared to those with MSD, which may be related to the characteristics of each disorder. Children with PD have difficulties in the process of speech representation (auditory and somatosensory), while those with MSD have difficulties in transcoding (planning and/or programming) and motor execution of speech, which also makes the representation process difficult, therefore, they are less frequent SSD but with greater commitments(1). Thus, these difficulties may be related to more serious alterations in the assessment of CAP.

When observing the data collected from parents, when answering the APDQ questionnaire, they did not observe changes in their children's auditory behavior and maintained the same score after the CAT intervention. This data is different from other findings(21,37) that use the auditory functioning scale - SAB(41) as a questionnaire and positively corroborate the same questions. Therefore, the questionnaire used in this research may have been less sensitive to the parents' perception due to the possibility of being longer and more complex for this audience or because they are already used to their children's complaints and perceptions.

The APDQ was recently validated for Brazilian Portuguese(34) and no data were found in the national literature to compare with those of the present study. No other inferences were made, as there is no published research that allows comparisons with the data from this study, indicating its originality.

Research was found relating CAP and AT to SSD, but more specifically to PD(18,20,21) and no publications with AT in children diagnosed with MSD in co-occurrence with CAPD.

Children undergoing speech therapy associated with Brief-CAT presented a significantly greater number of acquired sounds and a lower number of absent sounds in the phonological system, and the severity of SSD was, on average, milder than in the cases of children on the waiting list. Thus, there was an impact on speech when speech therapy was associated with Brief-CAT.

The Brief-CAT reflected in the speech results of subjects with PD, presenting better results than those with MSD, regardless of whether or not they received speech therapy. The results were significant for children with PD in terms of a lower number of absent sounds, a higher number of sounds acquired in the phonological system, in addition to a higher percentage of correct consonants and, consequently, a lower severity of the SSD. Therefore, the Brief-CAT impacted speech results according to the type of SSD.

The effect of Brief-CAT is not sufficient without speech therapy to promote a change in the phonological system of the sample group. A study that adopted a non-linguistic auditory intervention approach in children with SSD showed that 12 AT sessions were not enough to promote the improvement of phonological skills(19). These data point in the same direction as those of the present study, since the phonological system of the sample group changed in relation to the variables number of sounds acquired, number of absent sounds and the severity of SSD because the children received speech intervention.

Different from the findings of this research, a study with CAT(20) tested the effectiveness of this training combined with speech skills in children with PD, including using the same software, and did not show any statistically significant difference regarding the results in the phonological system of the subjects after the intervention.

In cases of PD, speech difficulties mainly refer to the representation (auditory and somatosensory) of speech processes, that is, the difficulty lies in the organization of the phonological patterns that must be acquired, according to the linguistic environment in which the child is inserted. PD therapy focuses on approaches that seek to establish new speech patterns in the child, aiming to reorganize the phonological system, with the expectation that these patterns will generalize to untreated targets or situations(49).

Possibly, the improvement in the phonological system of the sample group with Brief-CAT associated with speech therapy is related to the characteristics of the therapeutic approach adopted.

In the treatment of PD, therapeutic approaches generally have a phonological focus, with some being more traditional, such as the Modified Cycles approach(50,51), which involves intervention based on altered phonological processes and the choice of target sound in pre-selected target words (production practice). During the sessions, auditory stimulation (auditory bombardment) is planned at the beginning and end of the session, interspersed with production practice. However, it is important to note that this therapeutic approach does not incorporate specific intervention in auditory skills, it only uses an auditory attention strategy.

Other therapeutic approaches for TF, such as Minimal Pairs/Minimal Oppositions, Maximal/Empty Set and Multiple(52-55), the emphasis of speech intervention is on phonemic contrast, on the acoustic differences of the sounds treated, and in the proposal by Bagetti et al.(52) there is an indication of expansion for the use of auditory stimulation (auditory bombardment) at the beginning and end of the session, interspersed with the practice of producing minimal pairs in target words. However, there is also no emphasis on auditory training.

In the case of MSD, the difficulties involve speech planning and programming (CAS and CD), and speech motor execution (SME), with speech therapy focusing on training speech motor gestures, using the Principles of Speech Motor Learning - PSML(56). Among the therapeutic approaches studied (DTTC - Dynamic Temporal and Tactile Cueing(57); PROMPT - Prompts for Restructuring Oral Muscular Phonetic Targets(58); and ReST - Rapid Syllable Transition Treatment(59-61)) there is no reference to a focus on training auditory skills in these proposals.

The data from the present study indicate that children diagnosed with PD may benefit more in speech development when undergoing CAT, when compared to children with MSD. This is because they only need to reorganize the speech representation process (auditory and somatosensory), which is at the cognitive/linguistic level, and occurs through feedback and feedforward. Children with MSD have difficulties in the transcoding process (planning and/or programming), or in the motor execution of speech, linked to the precision of speech production and consistency. Thus, cases of MSD are more serious due to difficulties in speech processes that need to be adapted until representation (auditory and somatosensory) is achieved at the cognitive/linguistic level(3). As previously pointed out, other inferences were not made, as there are no published data that allow comparisons with the data from the present study, since no publication was found with AT in children diagnosed with MSD in co-occurrence with CAPD.

Based on the data obtained in this study, the importance of assessing CAP related to children with SSD becomes evident, as all subjects in this research confirmed alterations in auditory skills in the behavioral assessment of CAP. Early diagnosis can be beneficial for the precise programming of therapeutic objectives in this population(44), both to help improve auditory skills and in the period of development of oral and written communication(46).

Gamified CAT options can be a viable and cost-effective alternative for speech clinics. Studies like this indicate that perhaps a greater number of sessions and this proposal combined with conventional speech therapy would have greater benefits for patients with different SSD.

This publication can be considered a pilot study, which points to interesting data for future reflections, because when reflecting on the design of an ideal research, this would be a randomized clinical trial, which is already in the planning phase. However, being able to carry out a study with therapeutic intervention and strict and careful methodological criteria such as those adopted in the present study brings interesting scientific contributions and directs future work by this group of researchers, as well as the work of other Higher Education Institutions.

## CONCLUSION

The Brief-CAT had an effect on the CAP skills of children with SSD, such as figure-background ability. All CAP tests showed an increase in the number of subjects who presented a profile from “altered” to “normal” after the intervention. The variables: “Speech therapy for SSD” and “type of Speech Sound Disorder” did not clinically influence the results of the behavioral assessment of CAP after the adopted intervention. Parents' perception in response to auditory behavior maintained the same results after the intervention.

The effects of Brief-CAT on the speech development of the children in this study allow us to conclude that speech therapy associated with CAT should be the most appropriate therapeutic indication, evidenced in the development of the phonological system and reduction in the severity of SSD. Children with PD showed better speech results compared to those with MSD.
