# Supplementary material for: Effect of hearing training on hearing and speech skills in children with speech sound disorders
Source: Codas. 2025 Feb 28;37(2):e20240008. doi: 10.1590/2317-1782/e20240008en (PMC11895834; doi:10.1590/2317-1782/e20240008en)
Supplement: Translation

# Translation: Efeito do treinamento auditivo nas habilidades auditivas e de fala em crianças com transtornos dos sons da fala

**DOI:** 10.1590/2317-1782/e20240008pt

**Authors:** Cristiane Dellinghausen Valim, Márcia Keske-Soares, Eliara Pinto Vieira Biaggio

**Affiliations:** 1 Universidade Federal de Santa Maria – UFSM - Santa Maria (RS), Brasil.

**Keywords:** Percepção Auditiva, Criança, Software, Estimulação Acústica, Distúrbios da Fala

## Abstract

**Objetivo:**

verificar o efeito do Treinamento Auditivo Computadorizado Breve (TAC-Breve) nas habilidades auditivas e de fala em crianças com Transtorno dos Sons da Fala (TSF).

**Método:**

participaram 14 crianças, de 6 e 9 anos, com diagnóstico de TSF. Todas crianças apresentaram uma ou mais habilidades auditivas alteradas na avaliação comportamental do Processamento Auditivo Central (PAC). Foram submetidas a seis sessões de TAC-Breve. Avaliou-se as habilidades auditivas e de fala e aplicou-se um questionário, pré e pós TAC-Breve. Realizou-se análise inferencial.

**Resultados:**

TAC-Breve teve efeito na habilidade de figura fundo para sons verbais. Os testes de PAC individualmente, apontaram evolução quanto ao número de sujeitos que modificaram seu perfil de “alterado” para “normal”, mesmo que nem todos tenham normalizado a avaliação. A terapia de fala prévia e o tipo de TSF não tiveram impacto nos resultados do PAC após o TAC-Breve. O questionário sustentou os mesmos resultados após intervenção. A terapia de fala quando associada ao TAC-Breve ofereceu maior possibilidade de adequação do sistema fonológico (diminuição de sons ausentes, maior produção de sons adquiridos e menor gravidade do TSF.) As crianças com transtorno fonológico obtiveram melhores resultados na fala ao realizarem o TAC-Breve associado, quando comparadas às com transtornos motores de fala.

**Conclusão:**

o TAC-Breve mostrou ser eficaz na melhoria da habilidade auditiva de figura-fundo em crianças com TSF. A terapia de fala associada ao TAC-Breve deve ser a indicação terapêutica mais adequada, pois proporciona maior evolução. O tipo de TSF influenciou na fala das crianças submetidas ao TAC-Breve.

## INTRODUÇÃO

A aquisição da fala ocorre ao longo da primeira infância e, em torno dos cinco anos, espera-se que a criança consiga produzir o padrão de fala, no qual está inserida socialmente. Quando tal aquisição não acontece como o esperado e alterações na sua produção são estabelecidas, como, por exemplo, trocas, omissões e/ou distorções de fonemas, tem-se os Transtornos dos Sons da Fala (TSF)(1,2). Tais transtornos são decorrentes de processos etiológicos, tanto genéticos como ambientais, associados ao neurodesenvolvimento(3). Nessa base desenvolvimental, os processos de fala, como a representação (auditiva e somatossensorial), transcodificação (planejamento e programação) e execução motora contribuem para o refinamento e/ou comprometimento da fala(1). O comprometimento da fala nestes processos específicos dá origem aos diferentes tipos de TSF.

Existem diferentes classificações dos TSF(4,5), porém a tipologia clínica mais usual atualmente é a que apresenta a distinção entre Atraso de Fala (ou Transtorno Fonológico - TF), Erros de Fala - EF (incluídos o Transtorno Fonético e os Erros Persistentes de Fala), e Transtorno Motor de Fala - TMF (e seus subtipos: Atraso Motor de Fala - AMF, Apraxia de Fala na Infância - AFI, Disartria Infantil - DI, e a combinação destes), diferenciadas quanto aos seus marcadores diagnósticos(1).

Estudos já publicados relacionam habilidades auditivas e habilidades linguísticas, especialmente em crianças com TSF(6-13), destacando que dificuldades na percepção auditiva de sons complexos, como os sons de fala, estão presentes nesta população. Isto reafirma a necessidade de avaliação auditiva, tanto em termos de sensibilidade quanto de percepção auditiva. Deste modo, como avaliação complementar à avaliação audiológica básica, utiliza-se a avaliação comportamental do Processamento Auditivo Central (PAC), para mensurar o desempenho relacionado à funcionalidade das habilidades auditivas centrais(14,15).

Na presença de alterações nos testes comportamentais de PAC tem-se como resultado o “atraso no desenvolvimento das habilidades auditivas centrais” (crianças com idades até 6:11,29 dias) ou diagnóstico de Transtorno do Processamento Auditivo Central (TPAC) (crianças acima de 7 anos). Em ambos, a conduta fonoaudiológica complementar é a estimulação das habilidades auditivas alteradas(15,16).

Dentre as estratégias de intervenção, o Treinamento Auditivo Computadorizado (TAC) é uma opção terapêutica interessante, pois o uso de *software,* além de ser referenciado cientificamente(6,9,17), é interativo e motivador. As estratégias de gamificação utilizadas contribuem para a manutenção de atenção/motivação e engajamento terapêutico(17). Assim, tal intervenção proporciona atividades de escuta desafiadoras (de forma aferente - processo *bottom-up*), recriando possibilidades adaptativas de escuta, assim como a necessidade de resposta motora (vias eferentes - processo *top-down*) por estas crianças(15).

Tem-se na literatura nacional, pesquisas correlacionando o TA aos transtornos de fala e linguagem(18,19), assim como nos casos de TF(20,21). Porém, a relação ao TA em diferentes TSF, incluindo os TMF, ainda se mostra carente de relatos, principalmente por ser abranger vários tipos de transtornos e sem um foco maior nas habilidades linguísticas.

Assim, por meio desta pesquisa buscou-se estudar uma abordagem terapêutica breve para crianças com alterações comportamentais nos testes de PAC que apresentam TSF.

Diante disto, o objetivo desta pesquisa foi verificar o efeito de um Treinamento Auditivo Computadorizado Breve (TAC-Breve) nas habilidades auditivas comportamentais e na fala de crianças com TSF.

## MÉTODO

Trata-se de um estudo longitudinal, prospectivo, quantitativo de cunho clínico-experimental, aprovado pelo Comitê de Ética em Pesquisa da Instituição sob o n° 68074623.0.0000.5346. Respeitou-se todas as diretrizes da Resolução 466/12, do Conselho Nacional de Saúde.

A amostra inicial contou com 26 crianças recrutadas em uma clínica-escola de Fonoaudiologia de uma Instituição de Ensino Superior. Sendo que destas, 14 concluíram todas as etapas da pesquisa. Cabe destacar que os participantes estavam em atendimento ou aguardavam na fila de espera para terapia de fala no serviço, e como critérios de inclusão neste estudo deveriam apresentar: diagnóstico prévio de TSF(1); limiares auditivos tonais dentro dos padrões de normalidade em ambas as orelhas e todas as frequências(22,23); em uma ou mais habilidades auditivas centrais alteradas na avaliação comportamental do PAC(14,16). Os critérios de exclusão foram estabelecidos quanto à: crianças com alterações do neurodesenvolvimento previamente diagnosticadas; com educação musical e/ou bilíngues; que tenham sido expostas a treinamento auditivo prévio; que não tenham completado o programa terapêutico proposto e/ou não realizado as reavaliações dentro do tempo pré-determinado para coleta dos dados.

Para estabelecer o diagnóstico dos diferentes TSF, todos os sujeitos foram submetidos às seguintes avaliações no setor de fala do serviço: Avaliação Fonológica (*software* INFONO - Instrumento de Avaliação Fonológica)(24); Avaliação de Vocabulário Expressivo (Teste Infantil de Nomeação - TIN)(25); Avaliação de Vocabulário Compreensivo (Teste de Vocabulário Auditivo - TVAud33)(26); Avaliação Dinâmica das Habilidades Motoras da Fala(27); e Avaliação Miofuncional Orofacial com Escores (AMIOFE)(28). A partir dos dados obtidos pelo INFONO, na etapa de nomeação espontânea, analisou-se a gravidade do TSF com os resultados do Percentual de Consoantes Corretas (PCC)(29,30), classificada em quatro níveis: Leve (PCC>85%); Leve-Moderado (PCC entre 65%-85%); Moderado-Grave (PCC entre 50%-65%); e Grave (PCC<50%).

Os TSF foram classificados pela tipologia supracitada(1), sendo que na amostra havia casos de: Transtorno Fonológico (TF); e de Transtornos Motores de Fala (TMF), com os subtipos Atraso Motor de Fala (AMF) e Apraxia de Fala na Infância (AFI). Salienta-se que os casos que apresentavam idade superior a nove anos foram classificados como com TSF Persistentes(31), seja de TF ou de TMF. Além de ser identificado o tipo de TSF dos participantes, a amostra foi distribuída em dois grupos: crianças com TSF em terapia de fala; e crianças com TSF em fila de espera.

Após, o grupo amostral foi convidado a participar do estudo e os pais e/ou responsáveis assinaram o TCLE e responderam às questões de anamnese. A seguir, foi realizada a avaliação audiológica básica (Meatoscopia, Audiometria Tonal Liminar de 250Hz a 8000Hz, Limiar de Reconhecimento de Fala com Figuras, Índice Percentual de Reconhecimento de Fala com Figuras, Timpanometria e Pesquisa de Reflexo Acústico Estapediano Contralateral). Todos deveriam apresentar limiares auditivos tonais dentro dos padrões de normalidade em ambas as orelhas e todas as frequências(22,23).

Na sequência foi realizada a Avaliação Comportamental do PAC, sendo realizados os seguintes testes: Teste de Memória Sequencial para Sons não-Verbais (TMSnV)(32); Teste Dicótico de Dígitos (TDD)(32); Teste de Detecção de Intervalo Aleatório (RGDT - *Random Gap Detection Test*)(33); Teste de Escuta Monótica por Sentenças (PSI - *Pediatric Speech Intelligibility*)(32). Todas deveriam apresentar ao menos uma habilidade auditiva alterada do PAC.

Além disso, foi solicitado aos pais que respondessem ao Questionário *Auditory Processing Domains Questionnaire* (APDQ)(34), composto por 52 perguntas divididas nos seguintes domínios: processamento auditivo (31 itens); atenção (10 itens) e linguagem (11 itens). Este questionário propõe-se quantificar, pela percepção dos pais, o comportamento auditivo das crianças e foi aplicado em sala de espera por uma avaliadora experiente. Os pais responderam às perguntas de forma objetiva em uma escala de quatro pontos. Deste modo, o comportamento auditivo das crianças foi avaliado, pontuando a cada questão: quatro pontos se o comportamento foi observado na maioria das vezes; três pontos quando observado com frequência; um ponto se observado algumas vezes; e zero ponto quando observado raramente.

Após estes procedimentos para a composição amostral, os participantes foram selecionados para a pesquisa. Como procedimento de pesquisa realizou-se um protocolo de intervenção denominado Treinamento Auditivo Computadorizado Breve (TAC-Breve). O TAC-Breve foi mediado pelo uso do *software* Escuta Ativa®(35), envolvendo 12 atividades para estimular as habilidades auditivas de interação binaural, figura-fundo, resolução temporal, padronização temporal, discriminação, integração e separação binaural.

O grupo amostral passou pelo TAC-Breve que foi realizado em seis sessões de treinamento, duas vezes na semana, com duração de 30 a 45 minutos cada, realizadas em sala silenciosa, utilizando computador da marca *Acer - Aspire 3 (A315-53-55DD)* e fone de ouvido supra-auricular da marca *Sennheiser*, modelo *HD 559*.

Em cada sessão, foram trabalhadas duas tarefas, com diferentes níveis de dificuldade (fácil, médio, difícil e insano denominadas assim pelo próprio *software*). Objetivou que cada criança acertasse um mínimo de 70% das tarefas para poder evoluir quanto ao grau de dificuldade, e assim, finalizar todas as atividades propostas. As duas atividades trabalhadas por sessão, seguiram a ordem de apresentação do *software* e na mesma sequência foram apresentadas ao grupo amostral.

O Quadro 1 foi elaborado para melhor visualização do protocolo do TAC-Breve realizado por meio do *Software* Escuta Ativa®(35), descrevendo as tarefas adotadas em cada sessão, suas características, principais habilidades auditivas envolvidas, e os níveis de dificuldade em cada uma delas.

**Quadro 1:** Protocolo de Treinamento Auditivo Computadorizado adotado: descrição de cada tarefa, principais habilidades auditivas estimuladas e níveis de dificuldade por tarefa do software Escuta Ativa®

Número de sessão	Tarefa	Características	Principais habilidades	Níveis de dificuldade
1º Sessão	Quantos Intervalos	Atividade onde o sujeito deve perceber os intervalos de silêncio entre os estímulos auditivos como, tons puros, músicas e frases.	Atenção auditiva e resolução temporal.	Fácil, médio, difícil e insano (entre tons, músicas e frases).
	Qual Som Ouviu	Duas palavras similares são apresentadas e o sujeito deve responder se elas são iguais ou diferentes. Neste último nível é inserido um ruído competitivo, aumentando a dificuldade da tarefa.	Atenção e discriminação auditiva.	Fácil, médio, difícil e insano.
2º Sessão	Audição e Atenção	São ouvidas duas palavras e deve-se identificar se elas estão de acordo com o enunciado da tarefa (relacionadas a consciência fonológica).	Análise e síntese auditiva, discriminação auditiva, atenção dividida, função executiva.	Fácil, médio, difícil e insano.
	Quantos Sons	Diferentes sons relativos a diferentes instrumentos são ofertados com a intenção de identificar a quantidade de estímulos apresentados.	Resolução temporal e atenção auditiva.	Fácil, médio, difícil e insano.
3º Sessão	Siga a Flauta	São apresentados sequências com diferentes padrões sonoros onde o sujeito deve reproduzi-las. Elas diferem em duração (curto - longo) e são tocadas ao som de uma flauta. A quantidade de estímulos sonoros em sequência varia de 3 a 5 sons.	Padronização temporal e memória auditiva.	Fácil (3 sons), médio (3 sons), difícil (4 sons) e insano (5 sons).
	Siga o Piano	São apresentados sequências com diferentes padrões sonoros e o sujeito deve reproduzi-las. Elas diferem pela frequência (grave - agudo) e são tocadas por um piano. A quantidade de estímulos sonoros em sequência varia de 3 a 5 sons.	Padronização temporal e memória auditiva.	Fácil (3 sons), médio (3 sons), difícil (4 sons) e insano (5 sons).
4º Sessão	Siga a Sequência	O sujeito ouve uma sequência de sons (como de animais) e deve pontuá-los no cenário apresentado, segundo as ordens solicitadas na tela.	Memória auditiva, memória operacional, integração do não-verbal ao verbal, função executiva.	Fácil, médio, difícil e insano.
	Bem na Mira	Estímulos dicóticos são apresentados e o sujeito deve identificar em qual orelha determinada palavra foi apresentada. Os sons alvo são números, palavras e expressões dicóticas.	Separação binaural e atenção auditiva.	Fácil (dígitos e palavras), médio (palavras, verbos e oposição), difícil (verbos, oposição e ditados) e insano (ditados).
5º Sessão	Esquerda – Direita	Duas palavras são apresentadas de forma dicótica, e o sujeito deve identificar quais palavras ouvidas em cada canal, selecionando-as entre várias opções similares apresentadas na tela.	Integração binaural e atenção auditiva.	Fácil, médio, difícil e insano.
	Binaural	Deve-se identificar a localização e a distância de estímulos que são apresentados em diferentes posições (direita ou esquerda / longe ou perto).	Interação binaural e atenção auditiva.	Fácil, médio, difícil e insano.
6º Sessão	Pegue se Puder (Faixa Bônus)	O sujeito deve seguir o deslocamento rápido de um item na tela, e clicar sobre ele.	Atenção visual, coordenação visuo-manual.	Fácil, médio, difícil e insano (com fases 1, 2 e 3).
	Siga o Ritmo (Faixa Bônus)	A partir de uma música pré-determinada, o sujeito deverá acertar o maior número de notas musicais ouvidas, pressionando setas de direção no teclado que se diferenciam por cores.	Processamento temporal, velocidade de processamento.	Fácil, médio, difícil e insano.

Como procedimentos de reavaliação, foram utilizados novamente os testes comportamentais do PAC, reaplicado o APDQ, assim como uma nova avaliação de fala foi realizada através da reaplicação do INFONO, em um período máximo de 30 dias após a realização do TAC-Breve, seguindo os mesmos padrões metodológicos de avaliação dos resultados da etapa pré-intervenção.

A etapa da reavaliação foi executada por profissionais habilitados do grupo de pesquisa, que não tiveram acesso aos dados da avaliação pré-intervenção e que não foram responsáveis pela aplicação do protocolo de TAC-Breve. Desta forma, pode-se concluir que este estudo tem uma análise de dados com cegamento, para reduzir ou eliminar o potencial viés de confirmação.

A etapa pós-intervenção teve como objetivo mensurar o efeito do TPC-breve (abordagem terapêutica complementar) em crianças diagnosticadas com TSF e a coocorrência das habilidades auditivas alteradas na avaliação do PAC.

Para análise dos dados foi realizado um estudo inferencial, no qual os resultados da avaliação comportamental do PAC, foram mensurados tanto em termos quantitativos quanto qualitativos. Os dados obtidos a partir da análise do APDQ foram analisados qualitativamente. Em relação aos dados da fala, estes foram analisados quantitativamente.

Além disso, as variáveis analisadas foram: estar ou não em terapia de fala para os TSF (em atendimento ou em fila de espera para a mesma) e quanto ao diagnóstico dos TSF (TF ou TMF).

Análises estatísticas descritivas e inferenciais foram feitas com o uso do *software Sas Studio*. Após testadas as pressuposições de normalidade (*Shapiro-wilk*), homogeneidade das variâncias (*Levene*) e independência dos erros (gráfico de resíduos), as respostas do PAC foram analisadas por meio dos testes *Tukey* e Teste não paramétrico de Fisher e as diferenças significativas foram declaradas quando o valor de p foi <0,05 e tendências foram consideradas quando 0,05 ≥ P ≤ 0,10. Além disso, a suficiência amostral foi comprovada pela análise de poder, na qual observou-se valores de probabilidade acima de 0,85 para as variáveis principais extraídas do conjunto total de dados.

## RESULTADOS

A amostra inicialmente recrutada, por conveniência, foi de 26 crianças. A partir dos critérios de elegibilidade e da disponibilidade de participação na proposta de intervenção pesquisada a amostra ficou composta por 14 crianças com TSF, 11 meninos (78%) e três meninas (22%), com idades entre 6 e 9 anos (média de 7,3 anos de idade).

Quanto ao tipo de TSF, nove crianças (64%) apresentavam diagnóstico de TF, sendo que quatro apresentavam TF Persistente, e cinco crianças (36%) apresentavam TMF. Destas últimas, os diagnósticos eram de AMF e AFI, sem nenhum caso de DI ou associação AFI e DI, e somente uma criança apresentava TMF Persistente.

A Figura 1 apresenta o efeito do TAC-Breve nos resultados dos diferentes testes comportamentais do PAC, mensurando as mudanças nas porcentagens de acertos pós-intervenção, independentemente do tipo de TSF.

**Figura 1:** (A-G) Efeito do TAC-Breve nas habilidades auditivas pré e pós intervenção em crianças com TSF: análise quantitativa (n=14)

**Legenda:** TDD IntBin = Teste Dicótico de Dígitos Integração Binaural; TDD SepBin = Teste Dicótico de Dígitos Separação Binaural; RGDT = *Random Gap Detection Test*; PSI = *Pediatrics Speech Intelligibility*; OD = Orelha Direita; OE = Orelha Esquerda; % = porcentagem; ms = milissegundos; Pré = resultados etapa antes da TAC-Breve; Pós = resultados etapa depois da TAC-Breve; ŧ = indica tendência entre o pré e pós intervenção; *indica diferença estatisticamente significante entre as etapas pré e pós intervenção Teste de *Tukey*

A Tabela 1 apresenta o efeito do TAC-Breve nas respostas das crianças (Normal ou Alterado) para cada um dos testes da avaliação comportamental do PAC, por meio do teste Teste Exato de Fisher.

**Tabela 1:** Efeito do TAC-Breve nas habilidades auditivas pós-intervenção em crianças com TSF: análise qualitativa (n=14)

Intervenção	Resposta		valor de p^1^
	Normal	Alterado	
**TMSnV**			
Pré	8 (57,1)	6 (42,9)	0,59
Pós	13 (92,9)	1 (7,1)	**<0,01** *
**TDD IntBin OD**			
Pré	2 (14,3)	12 (85,7)	<0,01*
Pós	7 (50,0)	7 (50,0)	0,99
**TDD IntBin OE**			
Pré	1 (7,1)	13 (92,9)	<0,01*
Pós	7 (50,0)	7 (50,0)	0,99
**TDD SepBin OD**			
Pré	6 (42,9)	8 (57,1)	0,59
Pós	8 (57,1)	6 (42,9)	0,59
**TDD SepBin OE**			
Pré	4 (28,6)	10 (71,4)	0,11
Pós	7 (50,0)	7 (50,0)	0,99
**RGDT (ms)**			
Pré	9 (64,3)	5 (35,7)	0,28
Pós	10 (71,4)	4 (28,6)	0,11
**PSI OD**			
Pré	13 (92,9)	1 (7,1)	<0,01*
Pós	14 (100,0)	0 (0,0)	-
**PSI OE**			
Pré	13 (92,9)	1 (7,1)	<0,01*
Pós	14 (100,0)	0 (0,0)	-

1 Probabilidade pelo Teste Exato de Fisher a 5% de significância;

* indica diferença estatisticamente significante entre as etapas pré e pós intervenção

**Legenda:** TMSnV = Teste de memória sequencial não verbal; TDD IntBin = Teste dicótico de dígitos Integração Binaural; TDD SepBin = Teste dicótico de dígitos Separação Binaural; RGDT = *Random Gap Detection Test*; PSI = *Pediatrics Speech Intelligibility*; OD = orelha direita; OE = orelha esquerda; ms = milissegundos; n = número amostral; Pré = resultados etapa antes da intervenção adotada; Pós = resultados etapa depois da intervenção adotada; EPM = Erro Padrão da Média

Na Tabela 2 foi analisado o efeito do TAC-Breve nas habilidades auditivas das crianças com TSF, em cada teste da avaliação comportamental do PAC, considerando se o sujeito amostral estava frequentando a terapia ou se aguardava em fila de espera, por meio do Teste de *Tukey*.

**Tabela 2:** Efeito do TAC-Breve nas habilidades auditivas em crianças com TSF com e sem terapia de fala prévia (n=14)

Respostas	Terapia		EPM^1^	valor de p^2^
	Sim (n=8)	Não (n=6)		
TDD IntBin OD (%)	73,69	47,15	6,22	**0,01** *
TDD IntBin OE (%)	68,43	61,27	9,13	0,59
TDD SepBin OD (%)	66,66	59,05	7,76	0,51
TDD SepBin OE (%)	59,19	57,89	8,34	0,91
RGDT (ms)	5,82	3,60	1,70	0,38
PSI OD (%)	99,31	83,06	5,82	0,08
PSI OE (%)	99,25	83,89	5,84	0,09

1 EPM = Erro Padrão da Média;

2 Probabilidade pelo Teste de Tukey a 5% de significância;

* indica diferença estatisticamente significante entre as etapas pré e pós intervenção

**Legenda:** TDD IntBin = Teste Dicótico de Dígitos Integração Binaural; TDD SepBin = Teste Dicótico de Dígitos Separação Binaural; RGDT = *Random Gap Detection Test*; PSI = *Pediatrics Speech Intelligibility*; OD = orelha direita; OE = orelha esquerda; % = porcentagem; ms = milissegundos; n = número amostral; Pré = resultados etapa antes da intervenção adotada; Pós = resultados etapa depois da intervenção adotada; EPM = Erro Padrão da Média; Sim = participando em terapia de fala para os TSF; Não = aguardava em fila de espera para terapia de fala para os TSF

Na Tabela 3 foi analisado o efeito do TAC-Breve nas habilidades auditivas de crianças, em cada teste da avaliação comportamental do PAC, considerando o tipo de TSF, ou seja, com TF (n=9) ou com TMF (n=5), também com o Teste de *Tukey*.

**Tabela 3:** Efeito do TAC-Breve nas habilidades auditivas de crianças com Transtorno Fonológico e Transtorno Motor de Fala (n=14)

Respostas	Tipo de TSF		EPM^1^	valor de p^2^
	TF (n=9)	TMF (n=5)		
TDD IntBin OD (%)	72,94	47,90	6,34	**0,02** *
TDD IntBin OE (%)	72,02	57,67	9,32	0,32
TDD SepBin OD (%)	85,02	40,69	7,91	<0.01*
TDD SepBin OE (%)	73,94	43,14	8,51	0,03*
RGDT (ms)	4,44	4,98	1,74	0,83
PSI OD (%)	99,21	83,16	5,93	0,09
PSI OE (%)	99,73	83,42	5,96	0,09

1 EPM = Erro Padrão da Média;

2 Probabilidade pelo teste de Tukey a 5% de significância;

* indica diferença estatisticamente significante entre as etapas pré e pós intervenção

**Legenda:** TDD IntBin = Teste dicótico de dígitos Integração Binaural; TDD SepBin = Teste dicótico de dígitos Separação Binaural; RGDT = *Random Gap Detection Test*; PSI = *Pediatrics Speech Intelligibility*; OD = orelha direita; OE = orelha esquerda; % = porcentagem; ms = milissegundos; n = número amostral; Pré = resultados etapa antes da intervenção adotada; Pós = resultados etapa depois da intervenção adotada; EPM = Erro Padrão da Média; TSF = Transtorno dos Sons da Fala; TF = Transtorno Fonológico; TMF = Transtorno Motor de Fala

No que se refere ao efeito do TAC-Breve na percepção dos responsáveis em relação ao comportamento auditivo infantil, tal mudança no comportamento auditivo não foi percebida pós intervenção, pela análise das respostas do APDQ (valor de p=0,99) realizada por meio do Teste Exato de *Fischer*.

A Tabela 4 apresenta o efeito do TAC-Breve na fala, ou seja, aspectos fonológicos do inventário fonológico geral e da gravidade do TSF, considerando a variável histórico de terapia de fala prévia (estar em terapia de fala ou estar na fila aguardando atendimento), por meio do Teste de *Tukey.*

**Tabela 4:** Efeito do TAC-Breve nos resultados de fala das crianças com TSF com e sem terapia de fala

Respostas	Terapia		EPM^1^	valor de p^2^
	Sim (n=8)	Não (n=6)		
Sons ausentes (n)	1,16	6,54	1,48	**0,03** *
Sons parcialmente adquiridos (n)	1,37	1,72	0,55	0,66
Sons adquiridos (n)	16,46	10,73	1,77	0,03*
Consoantes corretas (%)	84,08	63,04	6,93	0,06
Gravidade^3^	1,53	2,34	0,23	0,01*

1 EPM = Erro Padrão da Média;

2 Probabilidade pelo Teste de Tukey a 5% de significância;

3 Gravidade: 1 = leve (mais de 85% de consoantes corretas), 2 = leve/moderado (entre 65 e 85% de consoantes corretas), 3 = moderado/grave (entre 50 e 65% de consoantes corretas) e 4 = grave (abaixo de 50% de consoantes corretas);

* indica diferença estatisticamente significante

Na Tabela 5, foi analisado o efeito do TAC-Breve na fala, quanto aos aspectos fonológicos do inventário fonológico geral e da gravidade do TSF, conforme o tipo de TSF (TF ou TMF), assim como as demais análises inferenciais com o teste de *Tukey*.

**Tabela 5:** Efeito do TAC-Breve nos resultados de fala de crianças com diferentes tipos de TSF (TF ou TMF)

Respostas	Tipo de TSF		EPM^1^	valor de p^2^
	TF (n=9)	TMF (n=5)		
Sons ausentes (n)	1,22	6,48	1,52	**0,04** *
Sons parcialmente adquiridos (n)	1,09	2,01	0,55	0,59
Sons adquiridos (n)	16,69	10,51	1,66	0,03*
Consoantes corretas (%)	87,04	60,08	7,07	0,03*
Gravidade^3^	1,36	2,50	0,25	<0,01*

1 EPM = Erro Padrão da Média;

2 Probabilidade pelo Teste de Tukey a 5% de significância;

3 Gravidade: 1 = leve (mais de 85% de consoantes corretas), 2 = leve/moderado (entre 65 e 85% de consoantes corretas), 3 = moderado/grave (entre 50 e 65% de consoantes corretas) e 4 = grave (abaixo de 50% de consoantes corretas);

* indica diferença estatisticamente significante

**Legenda:** TSF = Transtorno dos Sons da Fala; TF = Transtorno Fonológico; TMF = Transtorno Motor de Fala

## DISCUSSÃO

O efeito do TAC-Breve foi eficaz para a melhora da habilidade de figura-fundo para sons verbais das crianças com TSF. Quando observado de forma qualitativa e individual, todos os testes de PAC apontaram evolução. Entretanto, cabe destacar que, apesar destes resultados, o TAC-Breve, nesta amostra, não teve o poder de adequar todas as habilidades auditivas avaliadas e não houve mudança na percepção dos pais quanto ao comportamento auditivo pós tal intervenção. As variáveis “Terapia de fala para os TSF” e “tipo de TSF” não influenciaram nos resultados da avaliação comportamental de PAC pós TAC-Breve.

O efeito do TAC-Breve nos resultados de fala foi importante, pois pôde-se inferir que a terapia de fala quando associada ao TAC-Breve ofereceu maior possibilidade de adequação do sistema fonológico, ou seja, diminuição de sons ausentes, maior produção de sons adquiridos e menor gravidade do TSF. Além disso, as crianças com TF obtiveram melhores resultados na fala ao realizarem o TAC-Breve associado, quando comparadas às com TMF.

Ao analisar o efeito da intervenção, observou-se melhora na habilidade auditiva figura-fundo para sons verbais, mensurado por meio das respostas de TDD (nas duas condições do teste e nas duas orelhas avaliadas) e PSI após o protocolo adotado de TAC-Breve.

Diferentes estudos apontam melhora nas habilidades auditivas após o treinamento auditivo, sendo este considerado uma opção de intervenção importante(17,36,37), visando a evolução por meio da neuroplasticidade cerebral(38,39).

Sobre o efeito do TAC-Breve na habilidade auditiva de figura-fundo para sons verbais, sabe-se que esta habilidade está associada à análise e síntese dos sons da fala na presença de sons competitivos. Assim como o presente estudo, em uma pesquisa realizada com crianças com diagnóstico de TPAC, sem associação do TSF, os atores também utilizaram um *software* para adequação das habilidades auditivas e observaram melhora significativa nesta mesma habilidade(40). Em pesquisa cuja amostra foi composta por crianças com TFS, o treinamento auditivo computadorizado também mostrou-se eficaz para melhorar essa habilidade(41). Cabe lembrar que habilidade de figura-fundo estaria relacionada à dificuldade em separar informações relevantes e atendendo ao foco auditivo do ruído competitivo(37). Assim, possivelmente o TAC-Breve tenha sido mais eficiente nesta habilidade, considerando as especificidades do *software* escolhido, mesmo que as questões temporais estejam mais presentes na estimulação proposta.

Os dados dos testes que avaliaram a habilidade auditiva de resolução temporal não tiveram diferença estatisticamente significativa após o TAC-Breve adotado no presente estudo. Tal resultado mostra-se diferente de outros estudos que observaram melhora nesta habilidade em crianças com TF após o TAC com a utilização do mesmo *software* (20,41) e/ou com outros softwares que também foram eficazes para adequar tal habilidade na presença de TPA(40,42).

Acredita-se que a falta de efeito do TAC nos resultados do RGDT evidencie a permanência da inabilidade em perceber auditivamente diferenças acústicas em razão do tempo, no grupo amostral deste estudo. Cabe destacar que possivelmente este resultado esteve diretamente relacionado à imaturidade na percepção de contrastes da fala(43). Isto é, tal dado pode ter relação com a alteração de fala dos sujeitos de pesquisa, uma vez que, os padrões temporais têm associação com a discriminação auditiva e os segmentos de fala, pois tal habilidade é crucial para a percepção de pequenas mudanças de intensidade, duração e frequência dos sons, tão importantes para a produção correta da fala(10,18,44).

No presente estudo é possível observar que em todos os testes, houve evolução quanto ao número de sujeitos que pontuaram melhor (de “alterado” para “normal”) após a intervenção, mesmo que ainda se caracterize o atraso ou transtorno de PAC. Os testes que apresentaram uma maior chance de modificação desse perfil foram os testes TMSnV, TDD IntBin e PSI, isto é, os testes com diferença estatisticamente significante. Os resultados dos testes RGDT, TDD SepBin mantiveram praticamente os mesmos após intervenção.

Assim, a evolução qualitativa dos sujeitos de pesquisa perante os testes da avaliação comportamental de PAC se mostra importante. Porém, como já referenciado, não houve uma melhora em todos os testes e nem uma adequação das habilidades auditivas de todo o grupo amostral, assim como em outros trabalhos nacionais que utilizaram diferentes *softwares* (6,20,21) e/ou outras modalidades de TA(37). Em geral, cada protocolo de TA foca em algumas habilidades auditivas e a generalização do efeito desta intervenção para outras habilidades ainda é algo que merece ser analisado com mais atenção.

Embora o tempo para realização de cada atividade e duração do programa de TAC, ainda não seja um consenso na literatura, hipotetiza-se que uma das justificativas para os resultados do presente estudo é o número reduzido de sessões. Na literatura tem-se o registro de protocolos de TAC com oito a 40 sessões(6,9,17,20,40,45). Optou-se por realizar um protocolo de TAC-Breve, com seis sessões, estudando assim uma proposta com aplicabilidade clínica e custo operacional menor. Tal escolha evitou inclusive a perda amostral ao longo da execução do protocolo de TAC-Breve. Cabe pontuar que todos os sujeitos estiveram presentes em 100% das sessões e a adesão à terapia é uma variável importante no processo de reabilitação. Sabe-se que quanto maior o tempo de treinamento, mais essas habilidades são reforçadas a fim de aprimorá-las(46), assim novas pesquisas com número maior de sessões pode ser uma alternativa no futuro, o que não invalida os dados aqui apresentados.

Ainda, em relação ao efeito do TAC-Breve nas habilidades auditivas, ao analisar as respostas obtidas com crianças em terapia de fala para os TSF e as que estavam em fila de espera, pode-se inferir que os resultados da bateria comportamental de PAC não foram influenciados por tal variável. Tal dado pode ser resultante do modelo terapêutico adotado na terapia de fala para os TSF, no qual não há de forma regular a estimulação de habilidades auditivas do PAC e pela falta de estimulação nas crianças da fila de espera.

Sabe-se que, no caso das crianças com diagnóstico de TF, em alguns tipos de abordagens terapêuticas há uma estimulação auditiva (anteriormente denominada bombardeio auditivo) que consiste em apresentar uma lista de palavras que é lida pelo terapeuta e ouvida pela criança com o objetivo estimular auditiva e visualmente a percepção do som-alvo em palavras que não estão sendo diretamente trabalhadas em sessão de terapia(47). No entanto, é importante notar que essa abordagem terapêutica não incorpora a intervenção específica em habilidades auditivas. Esta estratégia refere-se à estimulação da atenção auditiva, uma das habilidades mais básicas na hierarquia das habilidades de PAC(15,48) e pode-se sugerir que tal conduta, não tenha sido tomada com padrão pelas terapeutas nesse público.

Para a variável tipo de TSF, os resultados apontam que a habilidade auditiva de figura-fundo para sons verbais é melhor em crianças com TF quando comparadas às com TMF. Além disso, as crianças com TF também apresentam, de forma numérica, melhores resultados na avaliação de PAC, quando comparadas às com TMF, o que pode estar relacionado às características de cada transtorno. As crianças com TF apresentam dificuldades no processo de representação (auditiva e somatosensorial) da fala, enquanto que as com TMF apresentam dificuldades de transcodificação (planejamento e/ou programação) e de execução motora da fala, o que dificulta também o processo de representação, portanto, são TSF menos frequentes mas com maiores comprometimentos(1). Assim, estas dificuldades podem estar relacionadas a alterações mais graves na avaliação de PAC.

Quando observados os dados colhidos com os pais, ao responderem o questionário APDQ, estes não observaram mudanças no comportamento auditivo de seus filhos e mantiveram a mesma pontuação após a intervenção de TAC. Tal dado se mostra diferente de outros achados(21,37) que utilizam a escala de funcionamento auditivo - SAB(41) como questionário e corroboram de forma positiva para as mesmas questões. Deste modo, o questionário utilizado nesta pesquisa, talvez tenha sido pouco sensível à percepção dos pais pela possibilidade de ser mais longo e complexo para este público ou por já habituarem-se às queixas e percepções de seus filhos.

O APDQ foi recentemente validado para o Português-Brasileiro(34) e não foi encontrado na literatura nacional dados para comparar com os do presente estudo.

Outras inferências não foram realizadas, pois não se tem pesquisas publicadas que permitam comparações com os dados deste estudo, apontando o ineditismo deste. Foram encontradas pesquisas relacionando o PAC e o TA aos TSF, porém de forma mais direcionada aos TF(18,20,21) e nenhuma publicação com TA em crianças com diagnóstico de TMF em coocorrência ao TPAC.

As crianças em terapia de fala associada ao TAC-Breve apresentaram significativamente maior número de sons adquiridos e menor número de sons ausentes no sistema fonológico, assim como a gravidade do TSF foi em média mais leve que nos casos das crianças em fila de espera. Assim, houve um impacto na fala quando a terapia de fala foi associada ao TAC-breve.

O TAC-Breve refletiu nos resultados de fala dos sujeitos com TF apresentando melhores resultados do que os com TMF, independentemente de terem recebido, ou não, terapia de fala. Os resultados foram significativos para as crianças com TF nos aspectos de menor número de sons ausentes, maior número de sons adquiridos no sistema fonológico, além de maior Percentual de Consoantes Corretas e, consequentemente, menor gravidade do TSF. Portanto, o TAC-Breve impactou nos resultados de fala conforme o tipo de TSF.

O efeito do TAC-breve não é suficiente sem a terapia de fala para promover uma mudança no sistema fonológico do grupo amostral. Estudo que adotou uma abordagem de intervenção auditiva não linguística em crianças com TF, apontou que 12 sessões de TA não foram suficientes para promover o aprimoramento das habilidades fonológicas(19). Estes dados apontam na mesma direção aos do presente estudo, uma vez que, o sistema fonológico do grupo amostral modificou-se em relação às variáveis número de sons adquiridos, número de sons ausentes e a gravidade do TSF porque as crianças receberam intervenção para a fala.

Diferente dos achados desta pesquisa, um estudo com TAC(20) testou a efetividade deste treinamento combinado às habilidades de fala em crianças com TF, inclusive utilizando o mesmo *software*, e não evidenciou diferença estatisticamente significativa quanto aos resultados no sistema fonológico dos sujeitos após intervenção.

Nos casos de TF, as dificuldades de fala se referem principalmente à representação (auditiva e somatossensorial) dos processos de fala, ou seja, a dificuldade está na organização dos padrões fonológicos que devem ser adquiridos, conforme o ambiente linguístico em que a criança está inserida. A terapia do TF se concentra em abordagens que buscam estabelecer novos padrões de fala na criança, objetivando a reorganização do sistema fonológico, com a expectativa de que esses padrões se generalizem a alvos ou situações não-tratadas(49).

Possivelmente, a melhora no sistema fonológico do grupo amostral com o TAC-breve associado à terapia de fala tenha relação com as características da abordagem terapêutica adotada.

No tratamento do TF, as abordagens terapêuticas, em geral, são de enfoque fonológico, onde algumas são mais tradicionais, como a abordagem de Ciclos Modificada(50,51) que envolve a intervenção baseada nos processos fonológicos alterados, e escolha de som-alvo em palavras-alvo pré-selecionados (prática de produção). Durante as sessões, está prevista a estimulação auditiva (bombardeio auditivo) no início e no final da sessão, intercalado com a prática da produção. No entanto, é importante notar que essa abordagem terapêutica não incorpora a intervenção específica em habilidades auditivas, apenas utiliza uma estratégia de atenção auditiva.

Outras abordagens terapêuticas para TF, como as de Pares Mínimos/Oposições Mínimas, Máximas/*Empty Set* e Múltiplas(52-55) a ênfase da intervenção de fala é no contraste fonêmico, nas diferenças acústicas dos sons tratados, sendo que na proposta de Bagetti et al.(52) há indicação de ampliação para uso de estimulação auditiva (bombardeio auditivo) no início e no final da sessão, intercalado com a prática da produção dos pares mínimos em palavras-alvo. No entanto, também não há ênfase em treinamento auditivo.

No caso dos TMF, as dificuldades envolvem o planejamento e a programação da fala (AFI e DI), e a execução motora de fala (AMF), sendo que a terapia de fala tem foco no treinamento do gesto motor de fala, utilizando os Princípios de Aprendizagem Motora de Fala - PAMF(56). Dentre as abordagens terapêuticas estudadas (DTTC - *Dynamic Temporal and Tactile Cueing* (57); PROMPT - *Prompts for Restructuring Oral Muscular Phonetic Targets* (58); e ReST - *Rapid Syllable Transition Treatment* (59-61) *)* não há referência de enfoque em treinamento das habilidades auditivas nestas propostas.

Os dados do presente estudo apontam que as crianças com diagnóstico de TF podem se beneficiar mais na evolução da fala ao realizarem o TAC, quando comparadas às crianças com TMF. Isto porque elas precisam somente reorganizar o processo de representação da fala (auditivo e somatossensorial), que é no nível cognitivo/linguístico, e ocorre através do *feedback* e *feedforward*. Já as crianças com TMF apresentam dificuldades quanto ao processo de transcodificação (planejamento e/ou programação), ou de execução motora de fala, vinculados à precisão da produção e consistência da fala. Assim, os casos de TMF são mais graves pelas dificuldades nos processos de fala que precisam ser adequados até atingir a representação (auditiva e somatossensorial) no nível cognitivo/linguístico(3). Como apontado anteriormente, outras inferências não foram realizadas, pois não se tem dados publicados que permitam comparações com os dados do presente estudo, uma vez que, não localizou-se nenhuma publicação com TA em crianças com diagnóstico de TMF em coocorrência ao TPAC.

A partir dos dados obtidos neste estudo, torna-se evidente a importância da avaliação de PAC relacionada às crianças com TSF, pois todos os sujeitos desta pesquisa confirmaram alteração nas habilidades auditivas na avaliação comportamental do PAC. O diagnóstico precoce pode ser benéfico para a programação precisa de objetivos terapêuticos nessa população(44), tanto para auxiliar no aprimoramento das habilidades auditivas, quanto no período de desenvolvimento da comunicação oral e escrita(46).

Opções de TAC com gamificação podem ser uma alternativa viável e econômica para a clínica de fala. Estudos como este apontam que talvez um número maior de sessões e essa proposta aliada à terapia de fala convencional, teria maior benefício para os pacientes com diferentes TSF.

Esta publicação pode ser considerada um estudo piloto, que aponta dados interessantes para reflexões futuras, pois quando refletido sobre o delineamento de uma pesquisa ideal, esta seria um ensaio clínico randomizado, a qual já está em fase de planejamento. Entretanto, conseguir realizar um estudo com intervenção terapêutica e critérios metodológicos rígidos e cuidadosos como os adotados no presente estudo trazem contribuições científicas interessantes e direcionam trabalhos futuros deste grupo de pesquisadoras, assim como o trabalho de outras Instituições de Ensino Superior.

## CONCLUSÃO

O TAC-Breve teve efeito sobre habilidades de PAC das crianças com TSF, como na habilidade de figura-fundo. Todos os testes de PAC apontaram evolução quanto ao número de sujeitos que apresentaram o perfil de “alterado” para “normal” após a intervenção. As variáveis: “Terapia de fala para os TSF” e “tipo de Transtorno dos Sons da Fala” não influenciaram clinicamente nos resultados da avaliação comportamental de PAC pós a intervenção adotada. A percepção dos pais em resposta ao comportamento auditivo, manteve os mesmos resultados pós intervenção.

Os efeitos do TAC-Breve na evolução da fala das crianças deste estudo permitem concluir que a terapia de fala associada ao TAC deve ser a indicação terapêutica mais adequada, evidenciada na evolução do sistema fonológico e redução da gravidade do TSF. As crianças com TF apresentaram melhores resultados na fala em relação às com TMF.
